# The complete mitochondrial genome sequence of the karst-dwelling crab, *Terrapotamon thungwa* (Crustacea: Brachyura: Potamidae)

**DOI:** 10.1080/23802359.2022.2070038

**Published:** 2022-05-05

**Authors:** Chutintorn Yundaeng, Chaiwat Naktang, Sonicha U-thoomporn, Nattapol Narong, Phakamas Phetchawang, Wirulda Pootakham, Rueangrit Promdam, Sithichoke Tangphatsornruang

**Affiliations:** a National Omics Center, National Science and Technology Development Agency (NSTDA), Pathum Thani, Thailand; b Princess Maha Chakri Sirindhorn Natural History Museum, Prince of Songkla University, Hat Yai, Thailand

**Keywords:** *Terrapotamon thungwa*, Potamidae, terrestrial long-legged crab, mitochondrial genome, phylogenetic tree

## Abstract

*Terrapotamon thungwa* is a new species of terrestrial long-legged crab discovered in a karst landscape of southern Thailand. Here, we report the first complete mitochondrial genome of this crab species. The mitochondrial genome size is 16,156 base-pairs (bp), including 13 protein-coding genes (PCGs), 22 transfer RNA (tRNA), and two ribosomal RNA (rRNA) genes. The AT and GC content of the mitochondrial genome sequence is 73.2% and 26.8%, respectively. Phylogenetic analysis with 26 crustacean species, based on 13 mitochondrial conserve genes, showed that *T. thungwa* was closely related to other freshwater crab species in the family Potamidae.

The freshwater crabs of the family Potamidae in Thailand are diverse. A new species, *Terrapotamon thungwa* Promdam Yeesin and Ng 2017 is only known in the karst area of Thung Wa District, Satun province and Palian District, Trang Province, southern Thailand. The Thungwa long-legged crab is a karst-dwelling crab. Moreover, it can be distinguished from congeners by its karst habitat and taxonomic identification, as reported by Promdam et al. (2017). The population of Thungwa long-legged crab has a low dispersal ability and a high risk of extinction so basic genetic information and phylogenetic analysis are important for biodiversity studies. In this study, we report the first complete mitochondrial genome of *T. thungwa.* Mitochondrial genome characteristics were explored and its phylogenetic relationships within the family Potamidae was reconstructed.

Adult *T. thungwa* specimens were collected from Thung Wa District, Satun province, Thailand (7°08′00ʺN, 99°47′30ʺE). Sample collection in this study was permitted by the Institutional Animal Care and Use Committee, Prince of Songkla University (project number P-19-52245). The specimen was deposited in the reference collection of the Princess Maha Chakri Sirindhorn Natural History Museum, Prince of Songkla University (PSU), Hat Yai, Songkhla, Thailand (https://nhm.psu.ac.th/permanent-staff-th/rueangrit-promdam-th/, Rueangrit Promdam: rueangrit.p@psu.ac.th) under the voucher number PSUZC-CRU-0087. The leg muscle tissue samples were collected from a male crab, snap-frozen in absolute ethyl alcohol and stored at −80 °C until further use. Muscle tissue was homogenized in liquid nitrogen for DNA extraction using QIAamp Tissue Kit (Qiagen, Hilden, Germany). The DNA library was constructed using MGIEasy FS DNA library kit and sequenced with MGISEQ-2000RS sequencer (MGI Tech, Shenzhen, China). All cleaned reads were assembled by MitoZ version 2.4 (Meng et al. 2019). The genome was then annotated with the MITOS web server (Bernt et al. 2013). Protein-coding genes (PCGs), ribosomal RNA genes (rRNAs), and transfer RNA genes (tRNAs) were confirmed using the NCBI Basic Local Alignment Search Tool (BLAST) (Altschul et al. 1990). The *T. thungwa* mitochondrial genome was 16,156 bp, containing 13 PCGs, 22 tRNAs, and two rRNAs. The mitochondrial genome sequence was submitted to GenBank under accession number MW697087. All PCGs used the canonical ATN initiation codons (nine with ATG, three with ATA, and one with ATT) and the typical stop codons (TAA or TAG) except for three genes (*CYTB, ND*5, and *COII*), using incomplete stop codons (T––). The lengths of tRNA-coding genes ranged from 51 to 73 nucleotides. The AT and GC content of the mitochondrial genome sequence was 73.2% and 26.8%, respectively. We also studied the phylogenetic relationships between *T. thungwa* and 25 other species from the Crustacea subphylum (phylum Arthropoda). The sequence alignments of 13 conserved genes (*ATP6*, *ATP8*, *COX1*, *COX2*, *COX3*, *ND1*, *ND2*, *ND3*, *ND4*, *ND4L*, *ND5*, *ND6*, and *CYTB*) were performed the best-fit amino acid substitution models using ModelTest-NG (Darriba et al. 2020). The phylogenetic tree was calculated by the maximum likelihood (ML) method using RAxML-ng (Kozlov et al. 2019) and neighbor-joining (NJ) method using MEGA X version 10.1 (Kumar et al. 2018) with 1000 bootstrap replications. Both NJ and ML trees showed that *T. thungwa* was placed in the family Potamidae. However, the structures of NJ and ML trees were different at the family level. The ML tree placed Potamidae as a closely related family with Mictyridae, Gecarcinidae, Sesarmidae and Ocypodidae, all of which shared a common ancestor with Varunidae (Figure 1). Whereas, the NJ tree placed Varunidae closely related with Gecarcinidae, Sesarmidae which shared a common ancestor with Ocypodidae, Mictyridae and Potamidae families. Previous studies on mitochondrial genomes of species in Potamidae and Mictyridae families (Naktang et al. 2021; Sonthirod et al. 2022) reported trees similar to our NJ tree. This study will help us to fulfill the *T. thungwa* knowledge gaps in other molecular analysis and taxonomy identification in further research.

**Figure 1.**
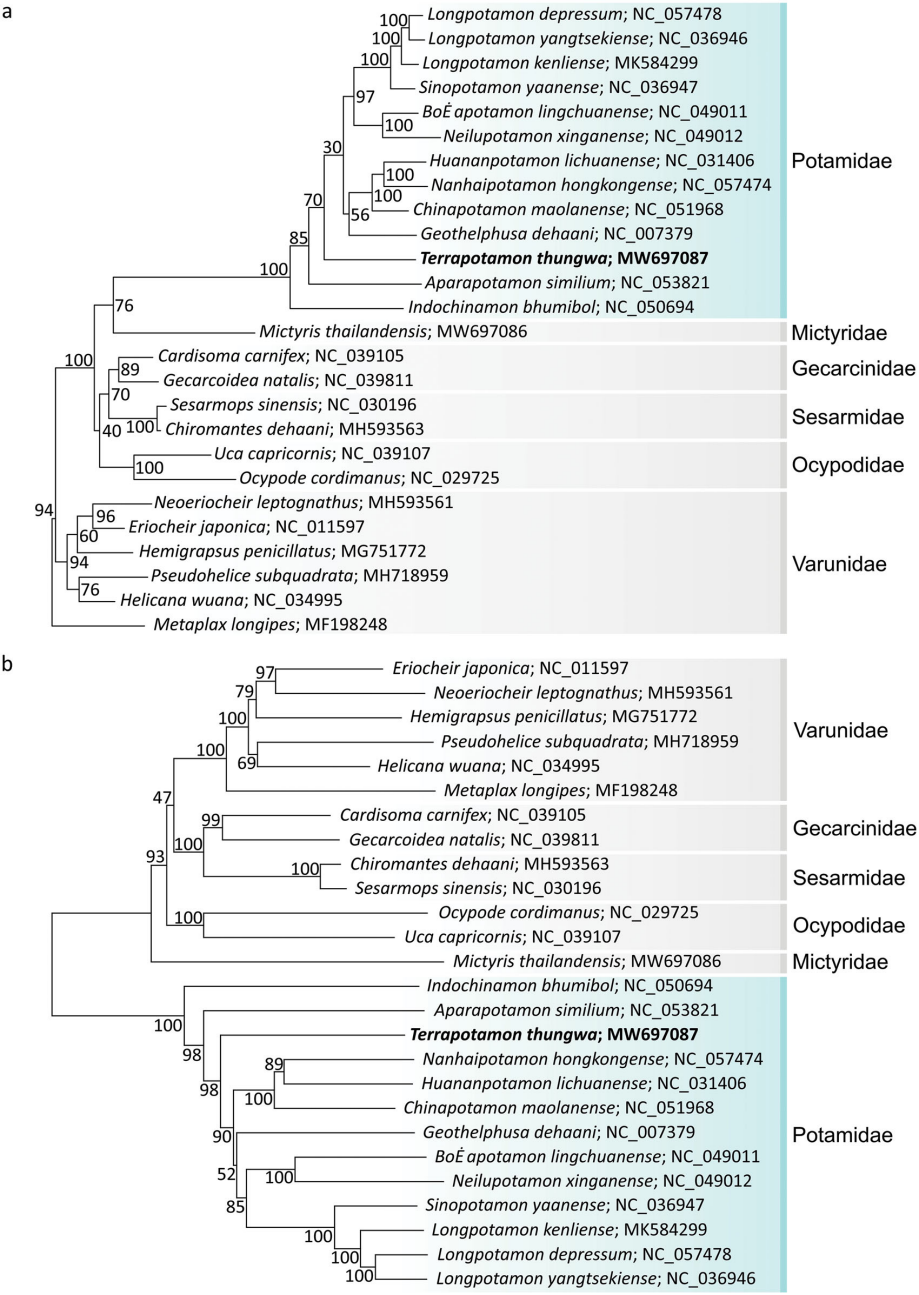
The phylogenetic tree of 26 species within the Crustacean class analyzed using maximum likelihood (a) and neighbor-joining methods (b) based on 13 protein-coding genes of the mitochondrial genomes. Numbers at the nodes are the bootstrap values. *Terrapotamon thungwa* is marked by bold letters.

## Authors’ contributions

WP and ST designed research study and obtained the funding. RP, SU, PP and NN performed laboratory work (sample collection, DNA extraction, library construction, and sequencing).CN performed bioinformatics analyses. CY wrote and revised the manuscript, and all authors reviewed it.

## Data Availability

The genome sequence data that support the findings of this study are openly available in GenBank of NCBI at https://www.ncbi.nlm.nih.gov/ under the accession no. MW697087. The associated BioProject, SRA, and BioSample numbers are PRJNA744328, SRR15056548 and SAMN20087478, respectively.
